# Outpatient Virtual Care Among People Living With and Beyond Cancer From Culturally and Linguistically Diverse Backgrounds in Australia: A Protocol for a Realist Evaluation

**DOI:** 10.1111/hex.70235

**Published:** 2025-03-18

**Authors:** Prince Peprah, Sagda Osman, Rebecca Mitchell, Ashfaq Chauhan, Ramya Walsan, Maryam Sina, Bronwyn Newman, Nadine El‐Kabbout, Jan Mumford, Emilie Francis‐Auton, Elizabeth Manias, Virginia Mumford, Kate Churruca, Michelle Moscova, Natalie Taylor, Craig Nelson, Alexander Cardenas, Robyn Clay‐Williams, Jeffrey Braithwaite, Reema Harrison

**Affiliations:** ^1^ Australian Institute of Health Innovation Faculty of Medicine, Health and Human Sciences, Macquarie University North Ryde New South Wales Australia; ^2^ Nafs Counselling Sydney New South Wales Australia; ^3^ Cancer Voices New South Wales Sydney Australia; ^4^ School of Nursing and Midwifery, Monash University Melbourne Victoria Australia; ^5^ Health ANSWERS (Health in ACT and NSW Education, Research and Services), Virginia Dr Bega New South Wales Australia; ^6^ Faculty of Medicine and Health, University of New South Wales Sydney NSW Australia; ^7^ Western Health Chronic Disease Alliance Victoria Western Health Melbourne Australia; ^8^ Department of Medicine – Western Health The University of Melbourne Melbourne Australia; ^9^ Health Infrastructure NSW, St Leonards New South Wales Australia

**Keywords:** Australia, CALD, cancer, models of care, realist evaluation, virtual care

## Abstract

**Background:**

Virtual care is increasingly being used to deliver outpatient cancer services, yet people from culturally and linguistically diverse (CALD) backgrounds can experience inequities in accessing these services. A range of complex and context‐specific factors impact the effectiveness of virtual care and equity in its use and outcomes. This study draws on the methodological principles of realist evaluation to provide contextual understanding and account of how, why and in what circumstances outpatient virtual care services work (or not) for people from CALD backgrounds accessing cancer services in Australia.

**Design:**

Realist evaluation, a theory‐driven approach, allows researchers to provide a nuanced understanding of how, for whom and why different interventions work (or not) under different circumstances. We propose an iterative and stakeholder‐driven four‐phase study design that is exploratory and sequential, following the Realist and Meta‐narrative Evidence Synthesis: Evolving Standards (RAMESES II) quality standards for realist studies. Phase 1 will generate the initial program theory from a realist synthesis of theories for how virtual care interventions are implemented into routine care and semi‐structured interviews with key stakeholders, including CALD service providers, service leaders and people with cancer and/or their carers who are from CALD backgrounds. Phase 2 will use semi‐structured realist interviews and focus group discussions with stakeholders and retroductive, theory‐driven realist analysis to test and refine the initial program theory. Phase 3 will validate the program theory with a small purposive participant sample outside those who participated in phases 1 and 2. The final phase will coproduce theory‐informed actionable recommendations and guidelines for effective virtual models of care implementation through interactive workshops with consumers, managers, service leaders and providers.

**Discussion:**

Knowledge of the contexts and mechanisms that produce optimal outcomes from virtual care is essential to guide the design, adjustment and implementation of virtual care models that provide equitable care outcomes for all patients. Outputs from this realist evaluation, including the program theory and actionable recommendations and guidelines, will inform policy and practice about implementing or adjusting virtual care models and policies or procedures in Australian healthcare to make them more accessible and equitable.

**Patient or Public Contribution:**

The conceptualisation and design of this study were developed with healthcare consumers from diverse cultural and linguistic backgrounds, healthcare providers and academics as part of a national project in Australia. Multicultural consumers who have lived experience of accessing cancer services contributed to the project's design as investigators and are coauthors of this protocol paper. Patients and the public are also represented as Project Steering Group members who will inform the data collection processes, development, and refinement of our program theory.

## 1 Background

The global cancer burden is growing, with an estimated 19.3 million new cancer cases and nearly 10 million deaths recorded in 2020 [1]. Australia exhibits one of the highest cancer incidence rates globally at 462.5 people per 100,000, which is mainly attributable to population increase, the ageing population and a national capability to detect cancers early [2, 3]. Among individuals aged 60–69 years, cancer is estimated to be responsible for approximately 47% of all deaths in Australia [2]. Irrespective of cancer mortality rates, cancer treatments and survival have improved over the last few decades [4, 5, 6, 7, 8]. Improvements in treatment and survival are partly due to greater access to screening services, investment in innovative treatments, and the use of technology, including outpatient virtual care modes of delivery, to improve timely access to care [7, 9, 10].

Culturally and linguistically diverse (CALD) groups experience a disproportionately higher burden of cancer and other chronic conditions compared to the wider population [11]. CALD groups or individuals identify with cultural backgrounds not predominant in Australia or speak languages other than English as their primary language, encompassing a wide range of communities, each with unique healthcare needs [12, 13]. People who are from CALD backgrounds are not a homogenous group, but they have diverse cultural and religious identities, norms, practices and languages, which also form part of their health and care delivery context [14]. Available evidence indicates that people accessing cancer services who are from CALD backgrounds experience significant disparities in access to cancer care services as well as poorer treatment quality and quality of life [15, 16].

Cancer care access and treatment disparities among CALD populations are attributed not only to biological factors but also to social, cultural and systemic issues [15]. Limited awareness of available healthcare options, compounded by language and cultural barriers, can impede timely medical attention and create difficulties in navigating unfamiliar health systems and services [15, 17]. On an institutional level, insufficient cultural competence (the ability to participate ethically, effectively and respectfully in personal and professional intercultural settings) within healthcare facilities exacerbates these challenges as many providers lack the necessary training and resources to effectively cater to the diverse needs of CALD patients [17, 18]. Collectively, these social, cultural, and systemic issues create substantial disparities in cancer care and treatment among CALD populations [15, 19].

Virtual models of care encompass a diverse range of modalities that leverage information technologies to deliver healthcare services remotely [20]. Although virtual care types are not the purpose of this study, these models can be categorised into two main types: synchronous care, which facilitates real‐time interactions between both clinician‐patient and clinician‐clinician to facilitate care delivery for patients via telephone or video consultations, and asynchronous care, such as remote patient monitoring, where patients use devices to collect and transmit health data to their healthcare providers for ongoing assessment [20].

Outpatient settings describe facilities such as a hospital or clinic where a patient receives care without being admitted and staying overnight. In these settings, virtual care models have demonstrated great potential in cancer care provision [21, 22]. Virtual care models have demonstrated greater effectiveness in resolving social and cultural barriers to care, improving accessibility, providing culturally competent care to CALD populations when compared to in‐person care. These impacts have been associated with increasing levels of satisfaction and cultural acceptance of virtual care models [23, 24]. One pilot study with culturally and linguistically isolated older Korean immigrants with depressive moods in the US found that tele‐counselling program implemented via videoconferencing on weekly sessions by Korean mental health counsellors led to a high level of program completion and overall satisfaction than in‐person sessions. Participants exhibited a significant reduction in depressive symptom severity shortly after completion of the program, with symptoms score remaining significantly lower at the 3‐month follow‐up [25]. Another study among culturally sensitive patients with diabetes in Alberta Cree Community reported that the introduction and incorporation of culturally sensitive programs and materials into tele‐ophthalmology program led to increased appointment attendance from 25% to 85%, patient satisfaction, trust towards the healthcare team and effective communication among providers and patients [26]. Thus, by promoting continuity of care and increasing flexibility and convenience while reducing the frequency of in‐person visits, these models can reduce healthcare inequities and help‐seeking in CALD patients [27, 28].

Notwithstanding the potential benefits of virtual models of cancer care among CALD populations, research into their clinical or patient outcomes has yielded mixed results. A recent scoping review analysing the equity across the cancer care continuum for CALD migrants living in Australia revealed varied outcomes, ranging from significant improvements to no observed difference and, in some cases, lower quality of life with virtual care use [15]. These mixed findings suggest that implementing virtual care for CALD populations may present unique challenges to health systems, services and providers due to many factors. Cultural and language barriers challenge the successful implementation of virtual care services for CALD communities [29, 30]. These challenges are further compounded by socioeconomic disadvantages such as low digital literacy among some CALD communities, limited access to devices and the Internet and the lack of support available for technology use in healthcare [17, 29, 31]. These factors underscore the need for culturally appropriate and competent virtual healthcare services [32, 33] to avoid widening the digital divide for CALD populations [34].

Evaluating the effectiveness and impact of virtual care is equally challenging due to the healthcare system's inherent complexity, the numerous interacting components and the stakeholders involved [35]. As these virtual care models continue to adapt and evolve as part of a complex healthcare system, their outcomes are influenced by the context within which they are embedded [35, 36, 37]. This complexity necessitates a nuanced approach to both the implementation and evaluation of virtual models of care. To date, virtual care researchers have rarely considered the complexities of the healthcare system and the contextual factors affecting the implementation of outpatient virtual models of care for CALD populations [38, 39, 40, 41, 42]. Realist evaluation provides a valuable framework to account for the intricacies of implementing and evaluating complex healthcare interventions such as outpatient virtual models of care for CALD groups [43]. By employing a realist evaluation approach, we seek to evaluate outpatient virtual care models for diverse CALD communities, focusing on the mechanisms of change and the contexts in which they operate. By conducting this evaluation, we will develop a theoretical understanding of the realities of how outpatient virtual models of care work and gain valuable insights into the nuanced contextual factors that influence the success or failure of outpatient virtual care for people of CALD backgrounds, particularly for the delivery of cancer care.

### 1.1 Overview of the Project Partners and Research Team

This realist evaluation is part of a larger program of research called the ‘Smarter Hospitals Project’, funded through the National Health and Medical Research Council Partnership Scheme Grant. This study involves partners across three Australian states, including New South Wales, Queensland and Victoria. These partners are knowledge users and active participants in the process and outcome of the realist evaluation; as such, their role will heighten the relevance of the research and uptake of the findings. The partners are relevant to this project because they were involved in the conception of the study as investigators and will continue to be involved throughout the research. For instance, some of the coauthors of this paper who provided useful comments and feedback to ensure that findings have important policy and practice relevance are from these partner organisations. Academic team members represent diverse disciplines and professional backgrounds, including social sciences, public health, health economics, psychology, allied health, nursing and medicine. Team members have extensive experience in implementation science and research, program evaluation, virtual care design and qualitative studies. Also, team members include those with experience of conducting realist synthesis and evaluation studies in Australia [44, 45] using methods relevant to the proposed research. Team members will bring these different perspectives to develop, test and refine the realist program theory and the study findings. The core team members meet fortnightly to review progress, establish procedures and milestones and maintain correspondence with the entire project team.

### 1.2 Research Objectives

This study aims to conduct a realist evaluation of outpatient virtual care interventions for people accessing cancer care services who are from CALD backgrounds. We specifically seek to:

a. determine the influence of varying contextual, structural, health system and organisational factors driving clinical and patient outcomes in outpatient virtual care provision;
b. build and refine a program theory that explains how differing contextual characteristics, strategies and mechanisms of change result in varying outpatient virtual care implementation outcomes (including acceptability, appropriateness, equity and uptake) and
c. coproduce actionable recommendations and guidelines to guide future virtual care models at policy and practice levels.

### 1.3 Research Questions

This realist evaluation seeks to answer the following broad question: ‘What works (the mechanism that make virtual care work) and does not work—how, why, for whom, to what extent and in what circumstances when providing virtual cancer care in outpatient settings for people who are from CALD backgrounds?’.

## 2 Methods

### 2.1 Realist Methodological Approach

Complexities in designing and implementing innovative care delivery models, such as virtual models of care within healthcare systems, create challenges in applying conventional research methods in their evaluation [46]. A realist approach is commonly adopted for the evaluation of complex, innovative health system implementation interventions and programs, such as virtual models of care [47], because it recognises that the process of change is not always linear and also the importance of context‐sensitiveness, in a complex adaptive system, such as healthcare [48, 49]. Due to this unique benefit, realist approaches have previously been used to analyse examples of successful and less successful transformation interventions to provide nuanced guidance for implementing large‐scale/system healthcare interventions in Australia and beyond [44, 50].

**Figure 1:**
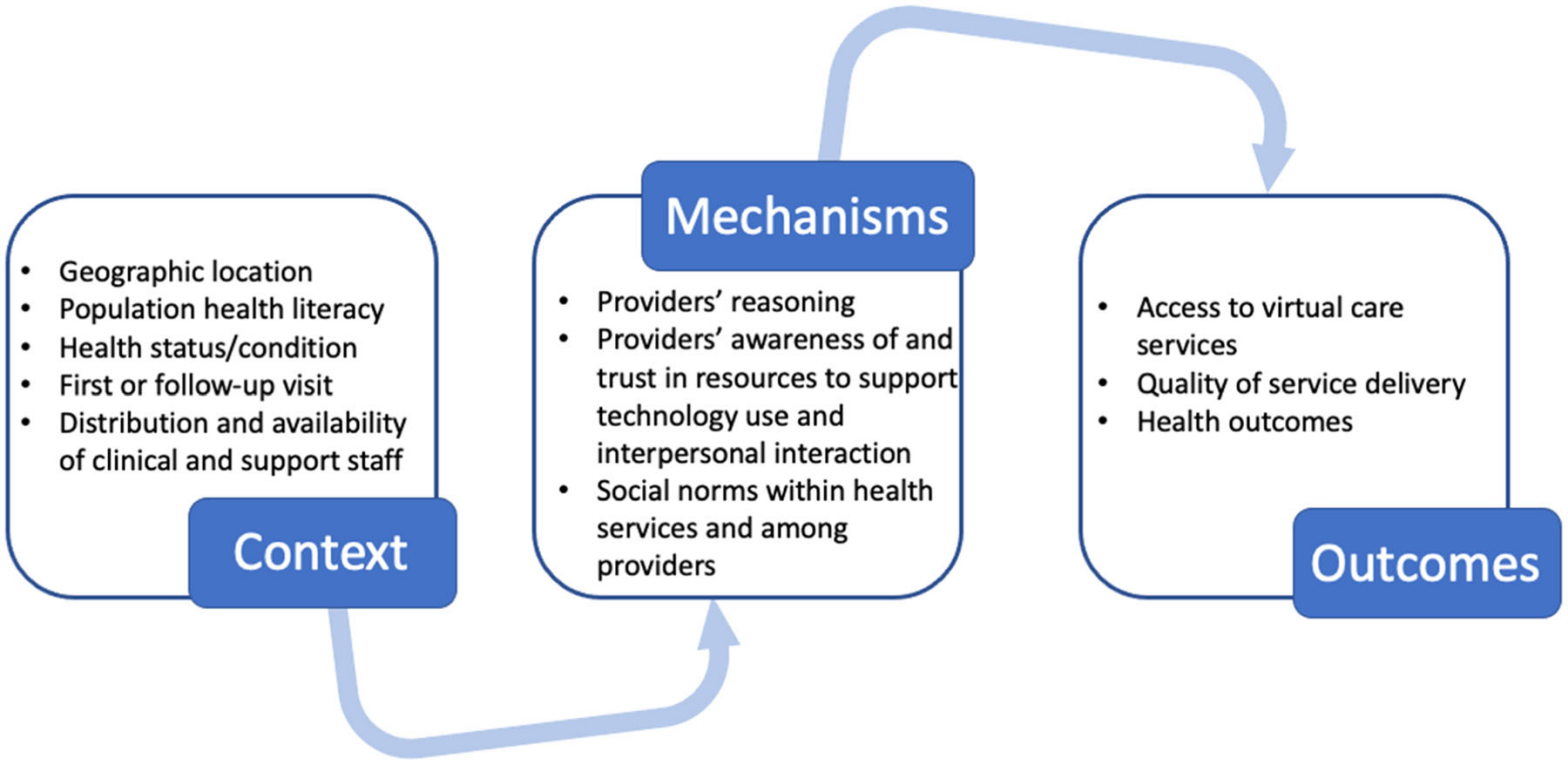
Prospective organising framework [54].

The outcome of our realist evaluation will be a program theory explaining how virtual care cancer services work in different settings and describing how any changes occurred because of the initiative(s) [51]. The program theory will be described in terms of the configuration of context, mechanism, and outcome, representing a contextually bound approach to causality: context + mechanism = outcome [52, 53]. See Figure 1 for an example of context‐mechanism‐outcome (CMO) configuration developed from initial work on clinicians’ experiences of virtual care models conducted by some of the authors of this protocol paper. Figure 1 forms the prospective organising framework for this realist evaluation study. It is acknowledged that this prospective framework is, at present, narrowly conceptualised based on findings from our previous work [54], but it is open to what may function as context within our CMO configurations during the various data analyses.

Context refers to the broad array of factors not necessarily linked to an initiative, which may facilitate or hinder the functioning of specific mechanisms [51, 55]. Context can transcend a setting or place; it constitutes anything that can influence how, why, and even whether the mechanism will work [46]. These include geographic location, population health literacy, health status/condition, distribution and availability of clinical and support staff, and the way they interact [56]. On the other hand, program mechanisms describe how it is that complex initiatives or interventions contribute to intended and unintended outcomes in specified contexts [57, 58]. The mechanism can include the emotional or cognitive responses of individuals experiencing a program implementation [55, 56]. Some authors argue that the mechanisms drive change rather than the program implementation itself [50, 51]. The resultant outcomes from the interplay between contexts and mechanisms could lead to either short or long‐term change, which can be desirable or undesirable [59].

Therefore, using realist evaluation, we seek to explain the interconnectedness among context, mechanism, and outcomes to develop a refined and validated program theory regarding how outpatient virtual care works (or does not work) for people from CALD backgrounds accessing cancer services and under what contextual conditions.

### 2.2 Scope and Population

For realist studies, the research aim should focus on specific processes or populations of recipients [60]. Thus, we will restrict our study to all people who are from CALD backgrounds, accessing outpatient cancer care services, either private, public or both. The study also focuses on CALD consumers who access cancer care services in urban or rural, or both areas. In this study, the term CALD is used to describe people who have a cultural heritage different from that of the majority of people from the dominant Anglo‐Australian culture [61]. CALD is a category recorded in the Australian health system. Efforts will be made to ensure that people living with cancer and their caregivers and families will be truly representative of CALD populations in Australia, as the study does not focus on specific CALD groups.

### 2.3 Our Study Design

We will employ a realist evaluation design that is exploratory and sequential [46, 55], following the Realist and Meta‐narrative Evidence Synthesis: Evolving Standards (RAMESES II), quality standards for realist studies [62]. We will draw on primary qualitative data and secondary evidence from realist synthesis and extensive experience working with health knowledge users, consumer groups and service providers. With the integration of multiple data, we seek to hypothesise, test and refine program theory about how and why outpatient virtual care interventions among people with cancer who are from CALD backgrounds lead to desirable or undesirable outcomes based on the impact of several factors, including those that are contextual such as geographical location, digital literacy and access and population health literacy [63, 64, 65].

### 2.4 Research Plan: Study Phases

**Figure 2:**
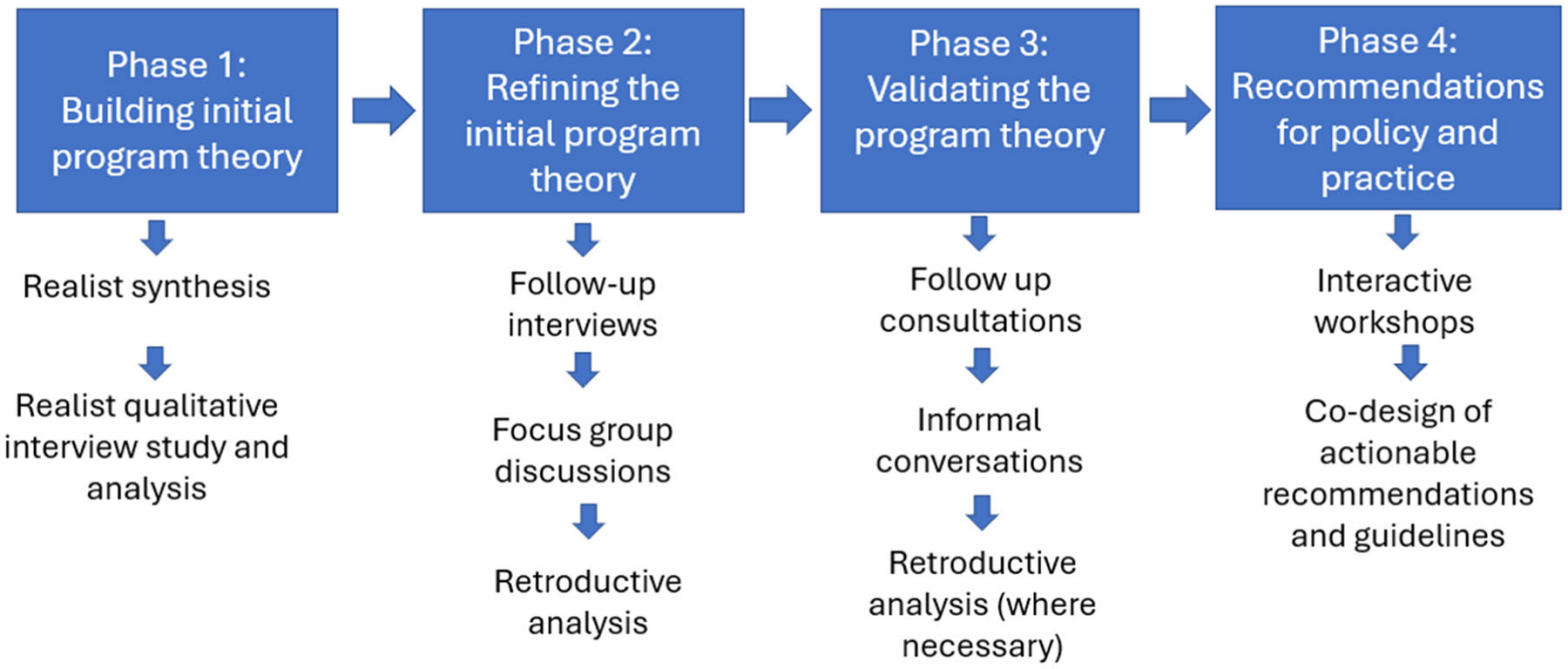
Four phases of the realist study and proposed process.

Figure 2 shows the study's four‐phase approach and summarises each phase's objective, primary outcomes, data sources and analysis approaches. Although the study phases are presented and described discretely, there will be some overlap between them in practice due to the iterative and cyclical nature of realist evaluations, which allows researchers to consult previous evidence as the understanding of the intervention deepens [66].

### 2.5 Phase 1: Building Initial Program Theory

In phase 1, we seek to build an initial program theory for how the provision of cancer services using virtual care in outpatient settings is both implemented and delivered (e.g., service providers, consumers, managers or the overall health system) to specifically explain why and how they do, or do not, work. We will gather evidence and collect thoughts, opinions, and ideas to generate hypotheses to develop the initial program theory.

#### 2.5.1 Methods

Two studies will be conducted in phase 1 [1]: a realist synthesis and [2] realist qualitative interviews.

##### 2.5.1.1 Study 1: Realist Synthesis

A realist synthesis of literature around outpatient virtual care interventions for cancer care services will be conducted. The purposes of this synthesis are to (1) explore the key concepts inherent in outpatient virtual care interventions for cancer services among people from CALD backgrounds using database searches (2) identify strategies used in their implementation; and 3) identify potential program theories that may explain strategies' mechanisms.

A detailed protocol for the realist synthesis will be developed and registered. Briefly, this realist synthesis will be guided by the work of Pawson et al. [64] and will follow guidelines stated in the Realist and Meta‐narrative Evidence Synthesis: Evolving Standards [53, 64]. As this synthesis aims towards building an initial program theory or theories of effectiveness of outpatient virtual care interventions for CALD populations with cancer, there are ongoing conversations with experts in the field and the Project Reference Groups regarding opinions on why outpatient virtual care interventions for delivering cancer care services for CALD populations work, who they work for, in what circumstances and why. We have also conducted a systematic literature search of academic and grey literature of outpatient virtual care interventions and their effectiveness for delivering cancer services for people from CALD backgrounds to get a general sense and understanding of available evidence around the topic. These conversations and preliminary literature searches will be used to guide the literature search for the synthesis.

Based on advice from a subject matter clinical librarian and information specialist and observations from the preliminary search, we will search online databases, including MEDLINE, Web of Science, Scopus, PsycINFO, Proquest Dissertation, CINAHL and EMBASE. In addition, web‐based searches will be conducted to retrieve grey literature, such as government and institutional reports from government and institutional websites and libraries, including Australian Universities' libraries, using search tools such as Google Advanced Search. Initial search terms and queries have been co‐developed with an experienced information specialist. The following types of keywords will be used to guide the initial searches (likely to evolve as the search develops). Population‐specific keywords include ‘CALD’, ‘ethnic minority’ and ‘cancer’. Intervention‐specific keywords include ‘outpatient virtual care’, ‘telehealth’ and ‘digital health’. The inclusion/exclusion criteria will be necessarily broad to ensure theory development is capable of taking in the widest range of evidence of theories [51]. For instance, we will include studies on people with cancer and their families and survivors who are from CALD backgrounds. We will include studies on healthcare professionals who use virtual care interventions with people with cancer and their families and survivors who are from CALD backgrounds. We will also include studies on interventions that involve virtual care modalities being used to deliver cancer services for a defined group of patients (specifically people with cancer and their families and survivors who are from CALD backgrounds). Studies that do not meet the above inclusion criteria will be excluded from the synthesis.

Information extracted will be categorised into context (such as settings, populations and intervention delivery), mechanisms (reasons behind the results) and outcomes (intended and unintended). These data will be recorded in NVivo 12 to promote a structured analysis. Two researchers (PP and SO) will independently extract these data. We will then collectively examine the data to detect patterns and develop compendium of explanatory factors observed in the included studies. The researchers will compare and discuss the identified factors that influenced the success or failure of virtual care for CALD populations being reported in the studies. Findings would be combined into a table detailing the number of studies proposing each mechanism and grouped by outcome. The two researchers will then jointly map recurrent patterns into explanatory CMO diagrams to illustrate how different factors interact [52]. The CMO diagrams will be discussed with a third member of the research team (EF‐A) to confirm consistent and logical development. Major findings will be synthesised into overarching themes, which are commonly referred to as ‘theories’ in the realist review approach [52, 64]. These ‘theories’ together with the findings of the Study 1 will form the initial program theory and inform the conduct of the subsequent phases towards tailoring and refining of the program theory.

##### 2.5.1.2 Study 2: Realist Qualitative Interview Study

To tailor the initial program theory, we will conduct semi‐structured interviews with stakeholders, including health consumers, CALD service providers (clinicians and translators/interpreters), service leaders and managers after the realist synthesis. We will conduct interviews with 15–20 health consumers (patients and/or their families or support persons) who are from CALD backgrounds with cancer. We will also conduct 10–15 interviews with outpatient virtual care implementors and evaluators such as CALD healthcare providers (including clinicians and translators), service leaders and managers from various health organisations such as hospitals in metro and regional areas within three Australian states. Although the final sample size will be determined based on emerging findings and data representation through an iterative data collection and analysis process [67], we aim to recruit up to 10 consumers and implementors and evaluators, each who have in‐depth knowledge about outpatient virtual care interventions design, implementation and use. Thus, overall anticipated minimum and preferable sample size for phase 1 is between 20 and 35 participants. This sample size is considered appropriate for a realist qualitative study among culturally diverse populations [45, 68].

A purposive sampling procedure supported by snowballing [14, 69, 70] will be used to identify and recruit health consumer participants with experience with virtual care in outpatient settings. The last author (R.H.) leads a consumer engagement network in health services called the CanEngage Network that focuses explicitly on equity issues. Through this study, > 30 consumer organisations will send recruitment information to distribute to members on our behalf. We will further engage Languages Other Than English (LOTE) Agency—a multicultural community engagement agency—that works with and connects bodies such as research teams with people who are from diverse cultural backgrounds. The LOTE Agency will invite health consumers to participate by promoting the study within the CALD communities, sharing the flyers with CALD consumers, and giving potential participants' details to the research team members. Moreover, our partner organisations will invite potential consumer participants by promoting the study to their consumers. CALD healthcare providers, service leaders, and managers will be sent invitation emails from partner organisations and other key government agency contacts to participate in an interview [71]. Social media platforms such as Facebook and LinkedIn will be used to reach additional participants.

Broad eligibility criteria have been set to ensure diverse participation. For health consumers, eligibility criteria for inclusion in the interviews include (1) people from CALD backgrounds who have ever or never used virtual outpatient cancer services in the past year before the interview; OR (2) informal carers of individuals from CALD backgrounds who have ever or never accessed virtual outpatient cancer care services in the past year before the interview; (3) living in either New South Wales (NSW), Victoria or Queensland and (4) have capacity and wish to participate in an interview While virtual care users will provide experiences regarding how virtual care is being used, non‐users will offer insights into why they are not using it. We will ensure a range of people who are from CALD backgrounds to enhance maximum variations in the sample and data. Eligibility for inclusion in the interviews for health professionals includes (1) Registered healthcare professionals who have provided virtual care to CALD patients in cancer services in the past 1 year before the interview; OR (2) directors, managers and other staff who have been involved in the development, implementation and delivery of virtual care services and (3) living in either NSW, Victoria or Queensland. This study will be conducted in these states because they are part of the most culturally and linguistically diverse Australian states [72]. Findings from the study could be useful for other states and beyond, particularly due to the commonalities in health and virtual care access challenges among people from CALD backgrounds [15, 32, 42].

In line with Ethics requirements, initial topic guides have been developed for healthcare consumers, clinicians and service leaders. Apart from sociodemographic questions such as age, gender and country of birth, some of the initial interview questions include: I'd like to hear about how you have used virtual care for healthcare at [service]? What are the things that make it easier for you to use virtual care? Has there ever been a time where virtual care did not go so well? If so, what was it that didn't work so well? (technology, communication). Overall, what is it about [service] that makes virtual care work [well, less well] for you?

However, based on the findings from the realist synthesis, interview questions will be reviewed and revised [73]. Such modifications will be submitted for Ethics approval before the commencement of data collection. Thus, the final interview questions will be determined by the findings from the literature review and synthesis. The final interview questions for health consumers will use plain language to provide interpretation for people who are from CALD backgrounds where required and for carers. Our consumer engagement network will further provide advice on the interview questions to ensure they are culturally appropriate. The interview questions will be pilot‐tested with our consumer engagement network to ensure their appropriateness. Interviews will be conducted in‐person, online, or at community libraries, staff workplaces, or a mutually convenient and agreed location, depending on the participant's preferences. Where a consumer prefers an online interview option, a brief orientation or training will be provided to the participant by the research team on using virtual platforms during online interviews before the actual interview, where necessary. Although interviews will be conducted by two experienced qualitative researchers (P.P., S.O.), consumer participants will be asked before the interview whether they would like to be interviewed by the researchers or a trained bilingual fieldworker. Also, participants will be asked before the interview whether they would like to be interviewed by an interviewer of the same gender to increase comfort and willingness to share information. The interviews will follow a realist approach [60, 68] to understand why variation exists about what takes place in natural settings, facilitating theories' development, testing, and refinement [43, 67].

We anticipate that each interview will last 30 to 60 min. We will use trained bilingual research fieldworkers or interpreters to offer language support in case the health consumers need it. Our existing consumer co‐facilitator network [74] comprises bilingual research fieldworkers who speak 11 languages. Members of this network have been trained in conducting qualitative research and interviews. These fieldworkers are bilingual and culturally competent, ensuring that they can engage with participants in a manner that respects their cultural norms and sensitivities. In case where members of the network cannot provide language support to a potential participant, professional interpreters will be used. The interview will start with an explanation of the purpose of the study and a brief description of the findings from the review and synthesis leading to the interview to provide participants with the context and background to the study. With the participants' consent, we will audio‐record interviews. Due to the iterative nature of realist studies, we may re‐interviewing participants to build our program theory as we gather additional insights [68, 73]. Thus, participants will be informed that the researchers may contact them for a short follow‐up interview to discuss findings. We will also reconfirm participants' wishes and capacity in any possible follow‐up interview.

Recordings that are not in English will be first translated into English by the trained bilingual fieldworkers for coding and analysis. Audio recordings in English will be transcribed by the researchers for coding and analysis. We will use NVivo 12 for data organisation and management. Multiple researchers will undertake the analysis continuously and concurrently alongside data collection. After the data collection, the researchers will read each transcript numerous times to achieve data immersion [45]. This will be followed by group‐work consensus building around CMOs and causal explanations [75]. The consensus‐building process will follow a realist dialogic approach, including team dialogues, discussions, meetings and conversations [45]. The program theory will be revisited and refined as an iterative process in light of the CMO data [46]. Consistency and transparency in the analysis will be achieved through structured analysis process and regular team meetings and discussions.

#### 2.5.2 Outcome

An initial program theory explaining contextual and mechanistic factors that could influence the successful implementation of outpatient virtual care for people accessing cancer services from CALD backgrounds.

### 2.6 Phase 2: Program Theory Refining/Testing

The iterative and flexible nature of realist methodology allows for testing the usefulness and quality of emerging evidence from one body of literature to another [64]. Phase 2 will test and refine our initial program theory generated in phase 1.

#### 2.6.1 Sample

The program theory refinement and testing will be conducted via interviews and focus group discussions. We will recruit at least 20 participants, selected from those who participated in the phase 1, including health consumers, CALD service providers (clinicians and translators/interpreters), service leaders and managers within and outside our partner organisations for the interviews. Twenty [20] participants including 15 consumers from diverse linguistic and cultural backgrounds and 5 providers from different health settings will be recruited for the focus group discussions.

#### 2.6.2 Methods

After generating the initial program theory in phase 1, an additional topic guides, based on the generated CMOs, will be developed to gather participants' perspectives on the initial program theory. In all, 20 semi‐structured interviews and three separate focus group discussions will be conducted. Each interview and focus group discussion will last between 30 and 90 min. The interviews and focus group discussions will capture narratives from health consumers and professionals to highlight implementation mechanisms within diverse contexts and any disparities in outcomes from outpatient virtual care identified in the prior interviews and literature review. These interviews and focus group discussions will also gather expert input into our initial theoretical framework. Participants who expressed interest to be contacted for further interviews in phase 1 will be contacted to take part in this phase. In an event that these participants cannot be recruited, we will rely on the same recruitment strategies outlined in the phase 1 study 3. Like phase 1, interviews and focus group discussions will be conducted in‐person, online, or at community libraries, or a mutually convenient and agreed location, depending on the participant's preferences. We will provide a brief orientation or training to the participant by the on using virtual platforms during online interviews before the actual interview, where consumers prefer an online interview option. The interviews will be conducted by the same interviewers who interviewed participants in phase 1. The focus group discussions will be moderated by the same research team members (P.P., S.O.). However, consumer participants will be asked before the interview and the focus group discussions whether they would like to be interviewed by the researchers or a trained bilingual fieldworker. Other processes outlined in phase 1 regarding language support, translation and transcription will be applied to this phase.

During the interview and focus group discussions, participants involved in phase 2 will be asked if they would like to be contacted again if further clarification or information is needed. A retroductive, theory‐driven realist approach will be applied to analyse the interview data gathered in phase 2 [76]. Retroductive analysis will combine inductive and deductive approaches to reveal the possible causal factors that explain the program theory. The interview and focus group discussion data from this phase will be used as evidence to refine the CMOs during retroduction. Thus, we will test the narratives and experiential information shared by the participants against the initial program theory by gathering opinions to support, refine and reject aspects of the theory [46].

#### 2.6.3 Outcome

A refined program theory of what successful outpatient virtual care among people with cancer who are from CALD backgrounds will look like in terms of the context in which virtual care works best and the mechanisms that support their intended outcomes.

### 2.7 Phase 3: Further Testing and Refining of Program Theory (Validation)

After refining the program theory and generating the CMOs in phase 2, we will validate the refined program theory with stakeholder groups in phase 3.

#### 2.7.1 Sample

The validation will be conducted with at least 15 participants, including five health consumers, professionals and service leaders each, selected outside those included in phases 1 and 2. The inclusion of people who are not participants of phases 1 and 2 is to ensure that the program theory is representative and generalisable. Efforts will be made to ensure the chosen participants represent broader participant groups engaged in the previous phases.

#### 2.7.2 Methods

To enhance the trustworthiness of the program theory and build a final synthesis narrative, further testing of the program theory and supporting evidence will be undertaken by conducting consultations with health consumers and health professionals outside the participants in phases 1 and 2. The same recruitment strategy in phase 1 will be adopted to recruit the participants for this phase. Interview data analysis will follow the same retroductive, theory‐driven realist analysis approach as phase 2. Where necessary, existing substantive theories such as candidacy [77] or normalisation process theory [78] will be used to further guide the analysis and develop the findings.

#### 2.7.3 Outcome

This phase will generate a validated program theory that comprehensively and practically explains what constitutes successful outpatient virtual care among people with cancer who are from CALD backgrounds. This validated program theory will represent the perspectives of health consumers, providers and service leaders about how, for whom, to what extent and in what contexts outpatient virtual care interventions work for people with cancer who are from CALD backgrounds.

### 2.8 Phase 4: Recommendations for Policy and Practice

In the final phase, we will present the findings of the realist study to health consumers, healthcare professionals, service leaders and managers and representatives from CALD communities and project partners to codesign actionable recommendations for the delivery of outpatient virtual care for people with cancer who are from CALD backgrounds.

#### 2.8.1 Sample

Purposively selected health consumers from diverse CALD backgrounds, healthcare providers and representatives of partner organisations.

#### 2.8.2 Methods

A codesign approach, which utilises coproduction processes, will be employed for this phase [79, 80]. The research team will conduct a series of face‐to‐face or virtual interactive workshops based on the findings from phase 3. Each workshop will be conducted for health consumers and professionals. Each workshop will have 6–10 members, excluding the research team. Participants in the workshops will include representatives of our partner organisations. They will be selected based on their experience and knowledge about virtual cancer care services particularly for people who are from CALD backgrounds. Each workshop will last approximately 2 h to allow for maximum and meaningful engagement of codesign members. We will adopt a flexible approach to participation to meet the needs of those participating in the workshop, which we have previously applied with many people who were in a range of points in their cancer journey [71, 81, 82]. The workshop content will be progressed in stages and participants can join in‐person or online. As part of the preparation for the workshop, individual participants' support needs will be identified and responded to ahead of commencement. Needs will also be reconfirmed throughout the workshop. The workshops will briefly discuss the findings from the various phases resulting in the validated program theory. Based on these discussions, priority areas will be identified for developing actionable recommendations and guidelines. Workshop with health professionals will be co‐facilitated by the first (P.P.) and fourth (A.C.) authors. In contrast, the health consumers workshop will be co‐facilitated by the first author (P.P.) and a trained consumer from a CALD background with lived experience, supported by bilingual fieldworkers relevant to the group members. Co‐facilitation with a trained healthcare consumer would help to address power differentials between, and the differing priorities of, facilitators and codesign members [74, 83]. Stakeholder consultations will be used to refine the recommendations and guidelines.

#### 2.8.3 Outcome

The outcome of this phase includes co‐designed actionable recommendations and theory‐based guidelines for designing and implementing virtual care models for people from CALD backgrounds. The recommendations and guidelines are intended to enhance practice in terms of outpatient virtual care delivery for people who are from CALD backgrounds, aiming to reduce health disparities and improve cancer care outcomes in these communities.

### 2.9 Patient and Public Involvement

The views of all partners have and will continue to be incorporated into the research. Diverse health consumers who are from CALD backgrounds have and will be actively participating as co‐investigators throughout the research, contributing to various aspects of the study such as recruitment and manuscripts development. Healthcare consumers involved in the study are remunerated financially for their specific contributions. A Project Steering Group involving healthcare consumers, clinicians and health policymakers has been established to inform and support the study's progress in all areas. Embedding strong patient and public involvement ensures the findings are clear and practically relevant to those designing and using outpatient virtual care for CALD communities.

### 2.10 Ethics, Data Storage and Protection

The Human Research Ethics Committee at Macquarie University has approved this study (Project ID 13038; Reference No:520231303852269). Where necessary, further ethics approval at the various health service sites would be obtained for the recruitment of healthcare providers and other staff. We will obtain informed written or verbal consent from participants for the study. Cultural considerations, such as language and community norms, will be central to our ethical approach, ensuring that participants from CALD backgrounds feel respected and understood throughout the study. Given the linguistic diversity of the target population, all consent materials, including information sheets, are written in plain language and translated into participants' preferred languages. Consent will be ongoing and/or renegotiated based on plans to follow up with participants for further interviews and confirmation of emergent findings. We will ensure participants' confidentiality and anonymity throughout the study, including during data gathering, management, analysis and reporting processes. All data from this study will be securely managed and protected, and only the researchers involved in the study can access study data. We will store data in hard copies, such as printed transcripts, in secured locked cabinets in the researchers' offices. We will store electronic data, such as audio recordings, on the Macquarie University OneDrive platform, which will be password‐protected and use multi‐authentication. All participants will be allocated an identification number, and the transcripts will be stored using these numbers. Participant contact details will be stored separately. De‐identified data could be made available upon request and with appropriate ethical approval in place as per open research principles. The data will be destroyed per ethical requirements 7 years after the project's completion.

### 2.11 Projected Outputs and Dissemination Plans

A program theory of outpatient virtual care will be developed, populated with evidence that explains ‘what works’ for virtual cancer care for people from CALD communities. An integrated knowledge translation approach [84, 85] will be adopted. We will seek the assistance of our participants and other stakeholders, including our study partners, in disseminating knowledge generated from the study to enhance applicability and impact.

Findings from the research will be presented in several formats and outlets. We will conduct workshops/meetings across Australia to promote the study findings and the co‐designed new guidelines. The main findings will be disseminated to inform policy and practice through our project partners, consumer groups, health organisations, and other relevant stakeholders. We will develop scientific conference presentations and academic articles to share our findings with the scientific and clinical communities. We will also develop infographic and digitally‐enabled outputs reporting the research outcomes, particularly the program theory, hosted on online platforms such as the Smarter Hospitals Project Webpage.

In addition to academic and professional dissemination, targeted community‐level dissemination will be a priority. Findings will be presented in culturally appropriate formats, including translated summaries, to ensure accessibility and relevance for CALD communities. We will collaborate with community leaders and organisations to facilitate the effective dissemination of these findings within CALD populations. These summaries will also be circulated via social media platforms like Facebook and LinkedIn.

### 2.12 Anticipated Strengths and Challenges

This realist evaluation will generate a detailed understanding of the relationships between contexts, mechanisms, and outcomes regarding the delivery of outpatient virtual care, allowing for the generalisability of findings to other contexts. A significant strength of our realist evaluation is the active engagement of stakeholders and consumers throughout the research process. By adopting this theory‐driven approach involving stakeholders such as health consumers and professionals, we will practically add to the knowledge base of virtual care models for CALD communities.

However, we will face some challenges. This planned realist study does not intend to collect primary quantitative data, thus, there will be a lack of primary quantitative data to test theories proposed in the qualitative data. Also, the time and resources required for realist studies are intensive due to their dialogical approach, which needs adequate planning and coordination [45]. The involvement of stakeholders and community members from the onset of the study could help address the anticipated timing and planning challenges.

Team members experienced in realist methodology will provide support and guidance throughout the process. For team members less experienced with these methods, realist synthesis and evaluation training will be undertaken. Thus, realist methodological support will be sought from the experienced team member and via external training mentorship as required at various stages throughout the project.

We anticipate that tensions could arise if different CALD populations and clinicians want different issues to be included in the guidelines and recommendations. Such tensions would be addressed through discussions and consensus building.

### 2.13 Timeline

This project forms part of a 5‐year national funded project, which began in 2023 and expected to be completed in 2027. The realist evaluation, which is one of the components of the overall project is being conducted for 30 months within the 5‐year project. The study 1 (realist synthesis) is expected to be completed within 11 months between February and December 2025. Study 2 (realist qualitative interviews) will be conducted within 6 months beginning from January to June 2026. Phases 2‐4 will be completed within 8 months, starting from July 2026 to February 2027.

## 3 Discussion and Conclusion

Health services interventions, such as integrating virtual models into routine care across the health system, are inherently complex and context‐sensitive. For populations with limited access to health services, particularly those from CALD backgrounds, understanding the factors that influence the successful implementation of these interventions is crucial to improving population health and reducing health inequities. This makes understanding their implementation effectiveness essential in achieving population health improvement, especially those with limited access to health services, such as people from CALD backgrounds, and reducing health inequities [86]. To the best of our knowledge, no evaluation of interventions such as virtual care for people living with cancer who are from CALD backgrounds using realist theoretical viewpoints exist, particularly in Australia. Thus, this four‐phase realist evaluation should identify context‐specific and mechanistic factors influencing outcomes and the successful implementation of outpatient virtual care for people with cancer who are from CALD backgrounds.

Recognising and understanding local contexts is vital for achieving the intended outcomes of health interventions, particularly in complex systems like healthcare [87, 88, 89]. Our study would identify factors across diverse studies and populations, allowing us to build a model for implementing outpatient virtual care for CALD communities. This model will be grounded in specific contexts but designed to inform broader applications, ensuring both relevance and scalability.

By involving health consumers, providers and key stakeholders, we are more likely to capture critical causal factors that might be overlooked in conventional evaluations. This engagement enhances the validity of our findings and ensures that the knowledge generated is directly applicable and impactful, facilitating the successful implementation of virtual care models for CALD populations. Our integrated knowledge translation approach synergises evidence generation with the active involvement of knowledge users, ensuring that the findings are both actionable and widely disseminated. This approach will facilitate the development of practical recommendations and guidelines that health policymakers, program planners, service managers, service leaders, and healthcare providers can use to inform, guide, and support decisions regarding virtual care planning, service delivery and quality improvement.

Through the development of program theory‐based recommendations and guidelines, our study will provide a deeper understanding of how the variable context, design and delivery of virtual care can influence outcomes for CALD populations. With active engagement and an implementation strategy, our findings will empower healthcare leaders to make informed decisions, ultimately contributing to more equitable healthcare access and improved outcomes for all, particularly those from CALD backgrounds.

## Author Contributions

**Prince Peprah:** writing – original draft, writing – review and editing, project administration. **Sagda Osman:** writing – original draft, writing – review and editing, project administration. **Rebecca Mitchell:** supervision, writing – original draft, writing – review and editing, funding acquisition. **Ashfaq Chauhan:** writing – original draft, writing – review and editing. **Ramya Walsan:** writing – original draft, writing – review and editing. **Maryam Sina:** writing – original draft, writing – review and editing. **Bronwyn Newman:** writing – original draft, writing – review and editing. **Nadine El‐Kabbout:** writing – original draft, writing – review and editing. **Jan Mumford:** writing – original draft, writing – review and editing. **Emilie Francis‐Auton:** writing – original draft, writing – review and editing. **Elizabeth Manias:** conceptualisation, writing – original draft, writing – review and editing, supervision, funding acquisition. **Virginia Mumford:** writing – original draft, writing – review and editing. **Kate Churruca:** writing – original draft, writing – review and editing. **Michelle Moscova:** writing – original draft, writing – review and editing. **Natalie Taylor:** writing – original draft, writing – review and editing, supervision, funding acquisition. **Craig Nelson:** writing – original draft, writing – review and editing. **Alexander Cardenas:** writing – original draft, writing – review and editing. **Robyn Clay‐Williams:** writing – original draft, writing – review and editing, funding acquisition. **Jeffrey Braithwaite:** writing – original draft, funding acquisition, supervision. **Reema Harrison:** writing – original draft, funding acquisition, writing – review and editing, project administration, supervision, conceptualisation, investigation.

## Conflicts of Interest

The authors declare no conflicts of interest.

## Data Availability

Data sharing is not applicable to this article as no new data were created or analysed in this study.
